# Grand Challenges in Molecular Medicine for Disease Prevention and Treatment Through Cyclical Innovation

**DOI:** 10.3389/fmmed.2021.720577

**Published:** 2021-07-15

**Authors:** Masuko Katoh, Masaru Katoh

**Affiliations:** ^1^ M & M PrecMed, Tokyo, Japan; ^2^ Department of Omics Network, National Cancer Center, Tokyo, Japan

**Keywords:** antibody-based biologics, artificial intelligence, clinical genome sequencing, COVID-19, FGF, reprogramming of tissue microenvironment, spatial biology, WNT

## Introduction

The coronavirus disease 2019 (COVID-19) pandemic, caused by severe acute respiratory syndrome corona virus 2 (SARS-CoV-2), has been affecting lifestyles and health care worldwide (West et al., 2020; Patel et al., 2021). Social distancing to avoid SARS-CoV-2 infection became a new normal during the COVID-19 pandemic. Scholars working in the fields of basic, translational and clinical medicine have adapted to COVID-19 turmoil through dynamic changes in their research methods and noticed some convenient aspects of remote work, teleconference and telemedicine (Ohannessian et al., 2020; Wang et al., 2021).

**FIGURE 1:**
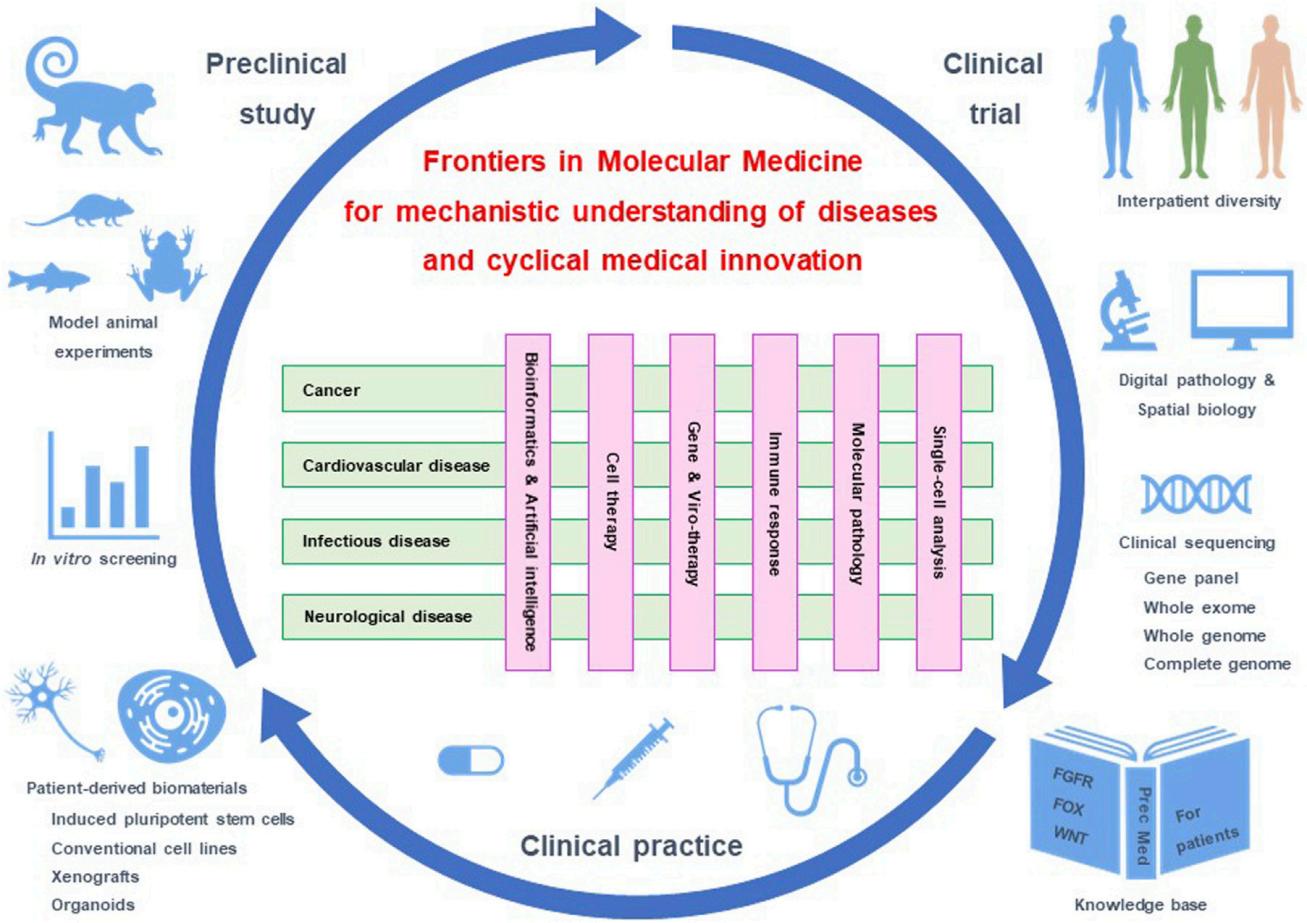
Molecular medicine and cyclical innovation. *Frontiers in Molecular Medicine* consists of four sections focused on diseases and six sections focused on methodologies. Preclinical studies using model animals and patient-derived materials, clinical trials using cutting-edge biomarkers, and clinical practice based on a knowledge base constitute a medical innovation cycle. Our journal addresses the understanding of disease mechanisms through cross-disciplinary interactions of scholars and the promotion of innovation cycles for the prevention and treatment of human diseases.

Molecular medicine is a field of medical sciences that addresses the mechanisms of human diseases (Figure 1). Preclinical studies using patient-derived cell lines, organoids and xenograft as well as animal models, such as monkey, mouse, Xenopus and zebrafish, are driving apparatuses for mechanistic understanding, target discovery and therapeutic optimization (Ramani et al., 2020; Rockx et al., 2020). Clinical trials are safety apparatuses to investigate the benefits and adverse effects of investigational diagnostics and therapeutics (Gyawali et al., 2019; Jung et al., 2021). Preclinical studies and clinical trials constitute a valuable core of molecular medicine to achieve innovation and improve medical practices (Figure 1).

There are three major obstacles that hinder cyclical medical innovation. Basic studies are not always recapitulated in clinical trials because of intrinsic biases of cell lines, engineered mouse models and human organoids; investigational drugs are not always approved for the treatment of patients owing to unknown on-target adverse effects; and approved drugs are not always beneficial for patients even after selection using companion diagnostics (Katoh, 2019; Katoh and Katoh, 2020). Integrative interactions of researchers in the fields of basic, translational and clinical medicine as well as those of cancerous and noncancerous diseases are essential to promoting innovation cycles in the healthcare and medicine sector.

Frontiers Media SA in Lausanne, Switzerland, is now launching a new journal, *Frontiers in Molecular Medicine*, to establish a platform of knowledge generation through timely publication of cutting-edge manuscripts and global networking of multidisciplinary scholars. The section structure of this journal will be briefly introduced, and then our perspectives on COVID-19, tumor heterogeneity and precision medicine will be discussed.

### Structure of *Frontiers in Molecular Medicine*

This journal comprises ten sections, each of which consists of Specialty Chief Editors, Associate Editors, Review Editors and Guest Associate Editors. Applications for Associate or Review Editors and proposals for Research Topics are welcome.

“Molecular Medicine and Cancer Treatment”, “Molecular Medicine for Cardiology”, “Molecular Microbes and Disease”, and “Molecular Mechanisms of Neurodegeneration” are disease-oriented sections that address human diseases, such as cancers, cardiovascular diseases, infectious diseases, and neurological diseases. Other common or rare diseases are also within the scope of our journal. Please consult editorial staff about the section to submit. A proposal of disease-oriented Section other than those mentioned above will be considered positively from viewpoints of innovativeness and networking potential of the applicant for its Specialty Chief Editor.

In contrast, “Bioinformatics and Artificial Intelligence”, “Cell Therapy”, “Gene and Viro-therapy”, “Molecular Mechanisms of Immune Response”, “Molecular Pathology”, and “Single Cell Analysis” are methodology-oriented Sections that address cutting-edge technologies, including antibody-drug conjugates (ADCs), bispecific antibodies, chimeric antigen receptor-modified T (CAR-T) cells, clinical bioinformatics, clinical sequencing, complete-genome sequencing, digital pathology, explainable artificial intelligence, liquid biopsy, oncolytic viruses, pluripotent stem cells, protein degraders, single-cell analyses, spatial biology, and telemedicine.

Cross-boundary interactions among disease- and methodology-oriented sections during the editorial process of submitted manuscripts as well as through arrangements of joint Research Topics are critical features of our journal for driving knowledge generation and horizontal innovation in the field of molecular medicine (Figure 1).

### COVID-19

SARS-CoV-2 enters and infects host cells through angiotensin converting enzyme 2 (ACE2) on endothelial cells, enterocytes and type II pneumocytes (Ziegler et al., 2020) as well as neuropilin-1 (NRP1) on endothelial cells, lung epithelium and olfactory epithelium (Cantuti-Castelvetri et al., 2020). COVID-19 patients present with fever, cough, fatigue, headache, hyposmia, hypogeusia, diarrhea and other symptoms, and some patients progress to severe conditions owing to respiratory, cardiovascular or cerebrovascular complications (Chen et al., 2020; Ellul et al., 2020; Nishiga et al., 2020). The case fatality rate of the general population with COVID-19 infection in the Worldmeters database is approximately 2.1% (3,331,763 of 160, 339, 530, as of May 12, 2021) (Worldometer, 2021); however, the rates of hospitalized COVID-19 patients in subgroups, such as the elderly, males, patients with diabetes, and the patients with hypertension and cardiovascular disease are much higher (Zhou F. et al., 2020; Grasselli et al., 2020).

Vaccines (BNT162b2, mRNA-1273, NVX-CoV2373, AZD1222 and Ad26.COV2.S) and therapeutic antibody cocktails (REGN-COV2) are SARS-CoV-2-targeted drugs for preventing infection or aggravation of COVID-19 (Polack et al., 2020; Ramasamy et al., 2020; Baden et al., 2021; Sadoff et al., 2021; Shinde et al., 2021; Weinreich et al., 2021). SARS-CoV-2 N501Y variants (B.1.1.7 in the United Kingdom) spread rapidly owing to increased affinity to ACE2 receptor, whereas SARS-CoV-2 N501Y/E484K variants (B.1.351 in South Africa) prone to escape antibody-mediated neutralization (Collier et al., 2021; Zhu et al., 2021). BNT162b2 vaccine showed 89.5 and 75.0% efficacies against B.1.1.7 and B.1.351 variants (Abu-Raddad et al., 2021). Because neutralization antibodies and T cell immunity are anti-viral mechanisms of dual wielding vaccines, vaccine-elicited T cell immunity might explain the benefits of vaccination against B.1.351 variants. Vaccine-based elimination is an optimal strategy to contain SARS-CoV-2 worldwide; however, global surveillance of SARS-CoV-2 variations and cyclic vaccinations targeting escape mutants might be necessary.

Repurposed drugs, such as anti-interleukin six receptor monoclonal antibodies (tocilizumab), broad-spectrum antiviral drugs (remdesivir) and corticosteroids (dexamethasone), are being applied to treat or ameliorate the symptoms of COVID-19 patients (Goldman et al., 2020; Stone et al., 2020; Tomazini et al., 2020), while an investigational ATR inhibitor (berzosertib) for the treatment of cancer patients with *ARID1A, ATM* and *SMARCA4* alterations based on synthetic lethal strategy (Williamson et al., 2016; Gupta et al., 2020; Yap et al., 2020) showed anti-SARS-CoV-2 activity in preclinical experiments (Garcia et al., 2021). Small-molecule compounds that block replication of SARS-CoV-2 would be game changers to end the COVID-19 pandemic in a few years.

In contrast, SARS-CoV-2 elicits versatile COVID-19 pathologies through direct infections, immunological responses and vascular damage in multiple organs or tissues. Because chronic persistent infection with hepatitis viruses and *Helicobacter pylori* cause liver cancer and gastric cancer, respectively (Ajani et al., 2017; McGlynn et al., 2021), and intracranial inflammation causes neurodegenerative diseases, such as Alzheimer’s disease and Parkinson’s disease (Heppner et al., 2015; Poewe et al., 2017), SARS-CoV-2 might also promote carcinogenesis or dementia through persistent chronic infection, dysregulated host immunity or vasculopathy. Epidemiological studies based on genomic analyses and mechanistic studies based on single-cell analyses should be conducted to elucidate the spatiotemporal profiles of COVID-19 pathologies in the future.

### Cancer Therapeutics and Tumor Heterogeneity

Whole-exome and whole-genome sequencing analyses on bulk tumors has revealed the genomic landscape of human cancers, including point mutations, fusions, gene amplifications and (super)enhancer alterations in cancer-related genes as well as numerous variants of unknown clinical significance (Rheinbay et al., 2017; Wong et al., 2020). Fusions of the *BCR* and *ABL* genes (*BCR-ABL*) in chronic myelogenous leukemia (Rosti et al., 2017), gain-of-function mutations of the epidermal growth factor receptor (*EGFR*) gene in lung cancer (Herbst et al., 2018), fusions of the fibroblast growth factor receptor 2 (*FGFR2*) gene with the *BICC1*, *KIAA1598*, *MGEA5*, *PPHLN1* or *TACC3* gene in cholangiocarcinoma (Katoh, 2019), amplifications of the *HER2* (*ERBB2*) gene in breast cancer (Oh and Bang, 2020) and exon 14 skipping mutations of the *MET* gene in lung cancer (Paik et al., 2020) are representative cancer drivers that are targeted by small-molecule inhibitors or antibody-based biologics in the clinic; however, there remain many cancer drivers that have not yet been successfully targeted in clinical practice.

For example, WNT signals are transduced to canonical and non-canonical pathways (Katoh and Katoh, 2007), and the canonical WNT signaling cascade is aberrantly activated in cancer patients owing to loss-of-function alterations in the A*PC, AXIN1, AXIN2, RNF43* and *ZNRF3* genes or gain-of-function alterations in the *CTNNB1* gene encoding β-catenin (Clara et al., 2020; Jung and Park, 2020). Investigational WNT signaling blockers of diverse therapeutic modalities, such as small-molecule compounds, peptide mimetics, antibody-based drugs and CAR-T cells, have shown striking benefits in preclinical studies but have not yet been approved for the treatment of cancer patients. Because the canonical WNT signaling cascade is involved in tumorigenesis as well as gastrointestinal, osteogenic and neuronal homeostasis (Katoh and Katoh, 2017), the therapeutic range of WNT signaling blockers might be too narrow for clinical application. Oncodevelopment signaling pathways with versatile functions in adult tissue homeostasis are hard targets for anticancer drug development.

Tumor heterogeneities, classified into interspecies, interpatient and intrapatient heterogeneity, are other obstacles to therapeutic development. Interspecies heterogeneity between human tumors and engineered mouse models is caused by species divergence in the coding regions and noncoding regulatory regions (Cornelissen et al., 2019; Pembroke et al., 2021). Interpatient heterogeneity or diversity is not completely recapitulated in human cell lines, xenografts and organoids owing to relatively small sample size and biases during the establishment procedure (Pauli et al., 2017; Bleijs et al., 2019). Engineered mouse models and patient-derived cell lines, xenografts and organoids are valuable tools for the screening and optimization of investigational drugs in preclinical studies; however, interspecies heterogeneity and interpatient heterogeneity might lead to the development failure of investigational drugs. A deep understanding of interspecies and interpatient heterogeneities with the aid of artificial intelligence and human intelligence would improve the success rate of drug development through orchestration of preclinical studies depending on therapeutic targets and drug moieties.

Intrapatient heterogeneity, further subclassified into intratumor and intertumor heterogeneities, is a hallmark of real tumors. Because antitumor immunity and therapeutic insults damage cancer cells but promote intratumor heterogeneity through acquired genetic alterations (Katoh, 2017; Marusyk et al., 2020), intratumor heterogeneity induces tumor evolution and subsequent intertumor heterogeneity of primary and metastatic lesions (Katoh, 2019; Vitale et al., 2021). Diagnostic genome sequencing based on a panel of approximately 500 cancer-related genes has been applied in the clinic to identify targetable cancer drivers (Katoh and Katoh, 2020; Yu et al., 2021); however, after targeted therapies, cancer drivers and dominant clones could be substituted by others to elicit therapeutic resistance. Temporal monitoring of cancer drivers for the optimization of targeted therapy is mandatory to improve the benefits and response rates of genome-based medicine.

The tumor microenvironment consists of slow-cycling cancer stem cells, proliferating cancer cells and noncancerous cells, such as cancer-associated fibroblasts, endothelial cells, immune cells and neurons (Lambrechts et al., 2018; Altorki et al., 2019). Paracrine and juxtacrine signaling networks within the tumor microenvironment, such as the WNT, FGF and Notch signaling cascades, maintain slow-cycling cancer stem cells and regulate tumor plasticity through omics reprogramming (Katoh, 2017; Katoh and Katoh, 2020). Complete or whole genome sequencing and single-cell analyses should be practiced in preclinical studies to decipher components of and networking within the tumor microenvironment for drug target discovery. Targeting aberrant features of the tumor microenvironment, such as immune evasion, matrix remodeling, metabolic adaptation and tumor angiogenesis, is an alternative strategy for cancer therapy compared with direct targeting of oncogenic drivers in cancer cells themselves (Valkenburg et al., 2018; Sahai et al., 2020).

## Precision Medicine in the Post-coronavirus Era

Precision medicine is defined as a medical system that utilizes clinical records, diagnostic imaging, laboratory tests, omics data and wearable device data for the prevention and treatment of human diseases (Collins and Varmus, 2015). Artificial intelligence or machine learning technologies are applied for target discovery and drug screening in preclinical studies as well as diagnostic medical devices in clinical practices of cardiology, endocrinology, gastroenterology, neurology, oncology, ophthalmology, pathology and radiology (Bera et al., 2019; Bi et al., 2019; Benjamens et al., 2020; Zhou Y. et al., 2020). Because risk of bias is a critical issue for black box-type artificial intelligence (Katoh and Katoh, 2020; Nagendran et al., 2020), artificial intelligence and human intelligence are both necessary to analyze multilayers of biodata and develop optimal diagnostics and therapeutics for the future implementation of precision medicine.

G protein-coupled receptors (GPCRs; ADRB1, GLP1R, HRH2, SMO and OPRM), receptor tyrosine kinases (RTKs; EGFR, FGFRs, HER2, MET, and VEGFR), intracellular enzymes (BRAF, CDK4/6, mTOR, PARP, and PI3K) and nuclear receptors (NRs; AR, ER and RAR) have been targeted using small-molecule compounds (Hauser et al., 2017; Katoh, 2019; Zhao et al., 2019; Roskoski, 2020; Wilcock and Webster, 2021), while RTKs (HER2), immune regulators (BCMA/TNFRSF17, CTLA-4, PD-L1, and PD-1), adhesion molecules (CLDN18.2 and NECTIN4) and miscellaneous transmembrane proteins (SLC39A6/LIV1 and TROP2) have been targeted using antibody-based biologics (Andrews et al., 2019; Bardia et al., 2019; Katoh and Katoh, 2020; Powles et al., 2021). Complete-genome sequencing based on long-read nucleotide sequences is superior to whole-genome sequencing based on short-read nucleotide sequences for the identification of novel biomarkers and therapeutic targets because of improved power for repetitive regions and segmentally duplicated regions (Logsdon et al., 2021; Rhie et al., 2021). Spatial omics approaches that reconstitute the tissue microenvironment at the single-cell level are necessary to decipher precise mechanisms of human pathologies and identify vulnerable targets to cure human diseases (Mathys et al., 2019; Travaglini et al., 2020).

Drug development for cancers and other diseases has been carried out by independent teams of experts in specific human diseases. The merits of disease-oriented approach include effective concentration of resources for drug development, such as clinical samples, patient-derived cell lines, organoids and xenografts as well as knowledge of experts in its specific field, whereas the demerits of such a traditional approach are overlooking or ignoring of the deleterious effects of drug candidates on other types of diseases. Artificial intelligence is applied for the discovery of drug targets and the development of therapeutics, while the human intelligence of multidisciplinary experts should be utilized to decide whether “druggable targets” are to be targeted.

Economic stimulus packages of governments and interest rate policies of national banks to support firms, health care systems and households have been alleviating the global economic crisis caused by the COVID-19 pandemic but might increase the financial burdens of societies in the future owing to unprecedented rise of government deficits and debt (International Monetary Fund, 2021a; International Monetary Fund, 2021b). Taking into account the unknown effects of COVID-19 and aging demographics, the implementation of precision medicine is mandatory for the improvement of human health care and the stability of the social insurance system in the post-coronavirus era.

## Conclusion

*Frontiers in Molecular Medicine* provides a platform of knowledge generation and horizontal innovation through the networking of scholars with diverse backgrounds, careers and ethnicities. We aim to advance the mechanistic understanding of human diseases for the development of novel diagnostics and therapeutics and contribute to the implementation of precision medicine through the promotion of cyclical medical innovation.
